# Tailored biomedical materials for wound healing

**DOI:** 10.1093/burnst/tkad040

**Published:** 2023-10-26

**Authors:** Wenhui Liu, Lihua Zu, Shanzheng Wang, Jingyao Li, Xiaoyuan Fei, Meng Geng, Chunlei Zhu, Hui Shi

**Affiliations:** Clinical laboratory, Affiliated Aoyang Hospital of Jiangsu University, 279 Jingang Road, Suzhou, Jiangsu, China; Jiangsu Key Laboratory of Medical Science and Laboratory Medicine, Institute of Stem Cell, Department of Laboratory Medicine, School of Medicine, Jiangsu University, Zhenjiang, China; Clinical laboratory, Affiliated Aoyang Hospital of Jiangsu University, 279 Jingang Road, Suzhou, Jiangsu, China; Department of Orthopaedics, Zhongda Hospital, Medical School of Southeast University, 87 Ding Jia Qiao Road, Nanjing, Jiangsu 210009, P.R. China; Jiangsu Key Laboratory of Medical Science and Laboratory Medicine, Institute of Stem Cell, Department of Laboratory Medicine, School of Medicine, Jiangsu University, Zhenjiang, China; Jiangsu Key Laboratory of Medical Science and Laboratory Medicine, Institute of Stem Cell, Department of Laboratory Medicine, School of Medicine, Jiangsu University, Zhenjiang, China; Jiangsu Key Laboratory of Medical Science and Laboratory Medicine, Institute of Stem Cell, Department of Laboratory Medicine, School of Medicine, Jiangsu University, Zhenjiang, China; Department of Orthopaedics, Affiliated Aoyang Hospital of Jiangsu University, 279 Jingang Road, Suzhou, Jiangsu, China; Clinical laboratory, Affiliated Aoyang Hospital of Jiangsu University, 279 Jingang Road, Suzhou, Jiangsu, China; Jiangsu Key Laboratory of Medical Science and Laboratory Medicine, Institute of Stem Cell, Department of Laboratory Medicine, School of Medicine, Jiangsu University, Zhenjiang, China

**Keywords:** Wound healing, Biomedical materials, Advanced materials, Tissue regeneration, Inflammation, Regeneration, Anti-bacterial, Cutaneous injury

## Abstract

Wound healing is a long-term, multi-stage biological process that mainly includes haemostatic, inflammatory, proliferative and tissue remodelling phases. Controlling infection and inflammation and promoting tissue regeneration can contribute well to wound healing. Smart biomaterials offer significant advantages in wound healing because of their ability to control wound healing in time and space. Understanding how biomaterials are designed for different stages of wound healing will facilitate future personalized material tailoring for different wounds, making them beneficial for wound therapy. This review summarizes the design approaches of biomaterials in the field of anti-inflammatory, antimicrobial and tissue regeneration, highlights the advanced precise control achieved by biomaterials in different stages of wound healing and outlines the clinical and practical applications of biomaterials in wound healing.

## Highlights

- This article reviews the design methods of biomedical materials in the field of anti-inflammatory, anti-bacterial and tissue regeneration.
- Biomedical materials have precise control in different stages of wound healing.
- Biomedical materials provide new insights into the treatment of cutaneous injury and the current challenges.

## Background

Normal wound healing is a complex physiological process with haemostatic, inflammatory, proliferative and remodelling phases. After wound formation, the coagulation system is activated and a local fibrin clot forms [1]. This is followed by an inflammatory phase during which monocytes transform into M1-type pro-inflammatory macrophages that engulf necrotic tissue and disintegrate neutrophil debris and bacterial products [2]. Subsequently, macrophages transform into M2-type anti-inflammatory macrophages, which release reactive substances that contribute to collagen synthesis and epidermal growth factor secretion, promote vascular endothelial cell proliferation and blood vessel formation, and stimulate wound healing into the proliferative phase. Fibroblasts also start secreting collagen, which eventually becomes a major component of the extracellular matrix, and wound healing gradually progresses to the remodelling phase [3–6]. These phases involve complex interactions between multiple cell types and growth factors and do not always occur in a logical order.

Burns, diabetes, infections and inflammation may cause a wound to heal slowly or become chronic nonhealing. Owing to the complexity of healing, re-establishing post-wound skin function remains difficult. Dressings, devices, medications, surgical interventions and biological approaches are currently used to treat wounds. Although the therapeutic efficacy of these approaches has been demonstrated, they are expensive and can result in adverse reactions such as rejection, infection and allergy. The main disadvantage is that these methods have a limited ability to initiate healing without stimulating subsequent reconstructive phases.

Therefore, innovative therapies should target specific events during the healing process to accelerate closure and restore healing tissues. Recently, Ag-modified polydopamine nanoparticles were embedded in a polysaccharide hydrogel-based network, resulting in excellent antimicrobial properties that can effectively promote wound healing [7]. Furthermore, nanomaterial-based wound dressings can effectively target different stages of wound healing using tannins, melanins and other nanomaterials with excellent antioxidant, antibacterial and photothermal properties.

In addition to nanomaterials, biomaterial-based wound dressings with biodegradability and biocompatibility are widely used at various stages of the wound-healing process [8–10]. Biomaterial-based wound dressings are available in various forms such as hydrogels, films, nanofibers, foams and patches. Because of their high biocompatibility, ability to encapsulate various types of cells and biomolecules, and controlled release in various wound models, biomaterials are promising candidates for wound healing applications. Hydrogels, for example, have favourable hydrophilic and haemostatic properties. The combination of nanomaterials with wound dressings has reactive oxygen-scavenging, haemostatic, antibacterial and proangiogenic properties that effectively promote diabetic wound healing [11–13].

Biomaterials have been used in medicine since the 1950s [14]. Most materials used at that time were biologically inert, but then it was discovered that these materials could interact with the body due to their biocompatibility [15]. In addition, some materials, such as sutures made of polylactic acid, can be degraded to harmless lactic acid and propylene glycol ester and slowly absorbed by the body [16]. With the development of biomaterials and engineering materials, research has shifted toward bioactive and intelligent materials, which can induce *in situ* tissue growth, promote tissue repair and respond to the internal environment [17–21].

Controlling infection and inflammation, as well as promoting tissue regeneration, are key strategies for managing favourable wound healing. Most chronic wounds are associated with infection and microbial film formation, which can lead to amputation, sepsis or even patient death if left untreated [20–25]. The immune system is important in the wound healing process, and the development of various immunomodulatory therapeutic strategies has received a lot attention in recent biomedical research. Immunomodulatory strategies typically promote wound healing by reducing inflammation and controlling the immune response after injury [1,9,26,27]. Furthermore, the exogenous addition of pro-proliferative active molecules, including growth factors, natural proteins, engineered peptides, stem cells and exosomes, can aid in wound healing.

**Figure 1:**
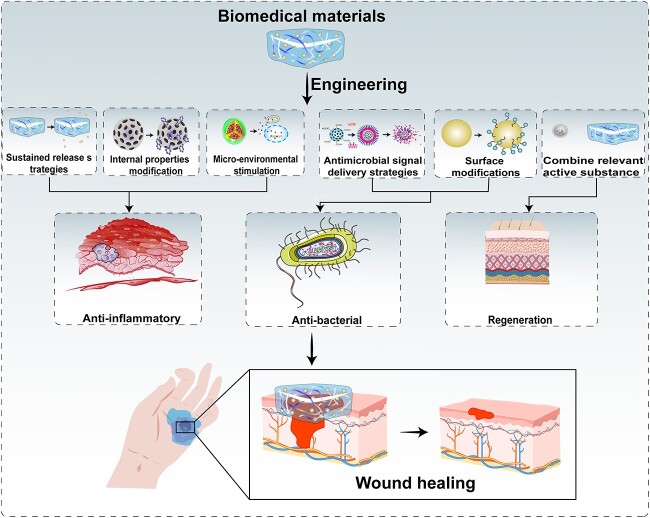
Schematic diagram of biomedical materials acting on different stages of wound healing

In most cases, specific components, such as nanomaterials, growth factors, cytokines and hydrogels, can stimulate each stage of the wound-healing process. Several review articles have discussed recent advances and current clinical strategies [9]. Although biomaterial-based approaches to promoting wound healing have been extensively studied, their applications have not been fully explored. In addition, there are many reviews on wound healing, but few on biomedical material design methods for different stages of wound healing. In this review, we summarize biomaterial design approaches based on antimicrobial, anti-inflammatory and tissue regeneration-promoting strategies. First, we discuss current biomaterial design strategies for antimicrobial, anti-inflammatory and tissue regeneration promotion, including the slow and sustainable release of relevant signals, modification of the biomaterial properties and mediation of the expression of relevant factors in the wound microenvironment. We also highlight studies on the application of engineered exosomes and synergistic biomaterials in tissue regeneration. Finally, we discuss how biomaterial personalization will increase clinical and translational potential in wound therapy (Figure 1).

## Review

### Biomedical materials for wounds

Biomedical materials can be classified into three categories based on their properties: metals, non-metallic inorganic materials and polymers. Metallic biomaterials are widely used in clinical applications owing to their excellent mechanical and regenerative properties [28,29]. They are widely used in prosthetic heart valves, vascular stents and bone repair implants. Recent metallic biomaterial research has focused on improving their properties using surface modification. For example, sandblasting, laser methods and electrochemical methods have been used on titanium microstructures and nanostructures to improve their interactions with cells [30–35]. In addition, metallic biomaterials, including Ag, Cu and Mg, have significant antimicrobial activity [36–39].

Non-metallic inorganic materials are classified into two main categories: crystalline and amorphous. Non-metallic and metallic elements are bonded together via covalent or ionic bonds to form bioceramic materials with immunomodulatory effects. For example, bioactive ceramics can regulate and convert M1-type macrophages into M2-type macrophages during the inflammatory phase of wound repair by creating a favourable microenvironment [40–42].

Bioactive glasses are another important class of non-metallic inorganic biomaterials that cause biological effects by binding to bone and releasing bioactive ions such as silicon, calcium and phosphate. Recently, biomaterials based on bioactive glasses have been optimized for various biological functions [43,44].

There are numerous polymer types with various properties, including hydrogels, nanomaterials, bioderived materials, natural polymers, synthetic polymers and biopolymers [45,46]. Hydrogels are 3D macromolecular networks that retain large amounts of water and have adjustable chemical and physical properties, biocompatibility, biodegradability, haemostasis, carrier properties and the ability to create a moist environment for wound healing, similar to the extracellular matrix. Hydrogels are widely used for wound-healing dressings [47,48].

Numerous multifunctional nanomaterials have been used in wound therapy in recent years to promote wound healing by taking advantage of their high reactivity, specific surface area, catalytic efficiency and loading efficiency. For example, iron tetroxide nanoenzymes have reaction kinetics similar to those of natural enzymes and can convert hydrogen peroxide into highly toxic hydroxyl radicals under mild conditions, resulting in antibacterial effects. Nanoenzymes have the advantages of low cost, easy synthesis, high stability and no external energy input compared with natural enzymes, and have become effective antimicrobial agents for wound treatment and bacterial infections [49–52].

Biologically derived materials mainly include the extracellular matrix and vesicles. Previous studies have demonstrated that mesenchymal stem cell (MSC)-derived extracellular vesicles have favourable repair effects on burns and diabetic wounds [53,54]. Natural polymers are biocompatible and biodegradable, but have weak immunogenicity. Examples include silk-fibronectin, chitosan, hyaluronic acid and alginate [55–60]. Synthetic polymers can exhibit greater processability and be endowed with specific biological activities through physical and chemical modifications. For example, arginine-glycine-aspartate peptides are commonly used for improving cell adhesion and can be immobilized on polymer molecules via chemical synthesis [61,62]. In addition, biopolymers such as peptides and nucleic acids have demonstrated significant potential in the field of biomaterials [63].

**Figure 2:**
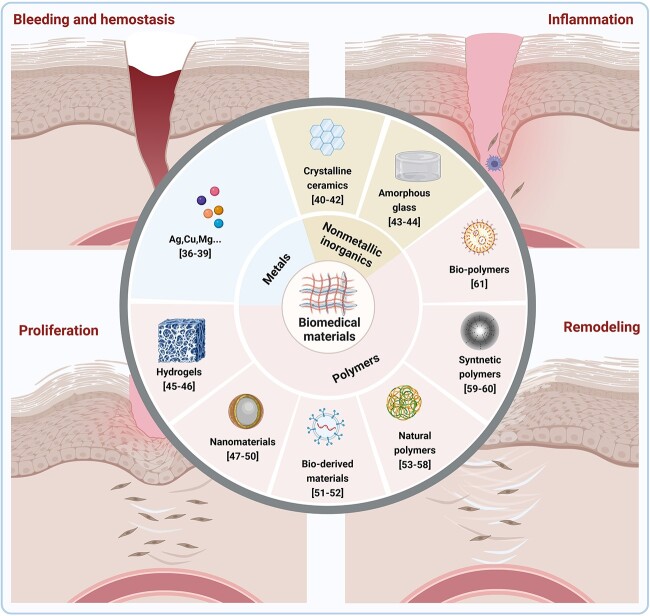
Classification of biomedical materials. Created with BioRender.com

Lacerations, burns and chronic refractory wounds are common clinical complications. Any disturbance in the wound-healing process, such as internal or external environmental irritation that results in chronic wound or prolonged healing, can pose a significant burden to clinical care and patients. Therefore, biomaterial design should be based on the principles of reducing healing time and improving healing efficiency. Anti-inflammatory, antibacterial and proliferation-promoting activities are three aspects of biomaterial design that need to be considered (Figure 2), and a detailed understanding of the mechanisms of action of different materials in these areas is important for designing materials to promote wound repair (Table 1).

**Table 1:** Examples of various biomedical materials used for wound healing

**Name**	**Design**	**Features**	**Potential roles in wound healing stages**	**Ref.**
EACPA	Slow release	Copolymerization of EGCG and APBA with acrylamide	Antibacterial, anti-inflammatory	[97]
Gel-Cip	Slow release	Cip loaded on PDANPs by hydrophobic and π–π stacking interactions	Antibacterial	[107]
HGGCD	Slow release	Gelatin, amoxicillin and carrageenan pre-gel infiltrated into anti-opalite scaffolds loaded with amoxicillin and VEGF	Antibacterial, promotes tissue regeneration	[161]
AH-3	Change its own characteristics	Synthesis based on a polyvinylpyrrolidone acrylamide 1-vinyl-3-butylimida zole and polyethylene glycol dimethacrylate method with cationic groups	Antibacterial	[109]
GP^Gel^	Change its own characteristics	Novel glycopeptides assemble into β-type lamellar gels by non-covalent bonding forces to mimic the skin’s extracellular matrix	Anti-inflammatory	[78]
starPEG-GAG	Slow-release, mediated trauma microenvironment-related signals	Electrostatic action binds chemokines MCP-1 and IL-8 and mediates the release of traumatic pro-inflammatory factors	Anti-inflammatory	[162]
Gel@fMLP/SiO_2_-FasL	Mediated trauma microenvironment-related signals	fMLP and SiO_2_-FasL for successful recruitment of neutrophils	Anti-inflammatory	[90]
SF/GA/ZN	Slow release	GA cross-linked with Zn^+^ and filament in protein GA signal	Anti-inflammatory	[73]
Cu5.4O@Hep-PEG	Slow-release, mediated trauma microenvironment-related signals	Amine-functionalized star polyethylene glycol -conjugated Cu5.4O heparin hydrogel, scavenging ROS, adsorbing MCP-1/IL-8	Anti-inflammatory	[163]
CIOS/Hydrogel/FGF	Slow release	Chitosan anti-opalite successfully loaded with fibroblast growth factor	Anti-inflammatory	[75]
bFGF-HDC@Fe_3_ O_4_	Slow release	Successful formation of surface-immobilized bFGF Fe_3_O_4_ nanoparticles	Anti-inflammatory	[72]
JNF-A	Alteration of its own properties, slow release	Successful synthesis of quaternized chitosan/polyvinyl alcohol loaded with curcumin and polygalactone	Antibacterial, anti-inflammatory	[87]
CS/PEG2	Slow release	Successful synthesis of chitosan hydrogels loaded with PEG2	Anti-inflammatory	[74]
G-DLPUs	Slow release	DLPU and GelMA form a dual network and successfully load L-Arg	Promotes tissue regeneration	[144]
ADAS/biomaterials	Slow release	Ethylene glycol chitosan and polyurethane Schiff base cross-linked loaded ADAS	Anti-inflammatory, promotes tissue regeneration	[164]
CBP/GMS@Cel&INS	Slow release	Phenylboronic acid grafted chitosan, ester bond and PVA hydroxyl group binding wrapped insulin and celecoxib	Anti-inflammatory, promotes tissue regeneration	[145]
HA@MnO_2_/FGF-2/Exos	Slow release	One-piece hydrogel with scheduled release of exosomes and fibroblast growth factor	Promotes tissue regeneration	[165]
FHE@exo	Slow release	Successful synthesis of peptide-based FHE hydrogels loaded with AMSC-exo	Promotes tissue regeneration	[137]
PUAO-CPO-EXO	Slow release	Antioxidant polyurethane combined with calcium oxide successfully loaded with AMSC-EXO	Promotes tissue regeneration	[150]
Gel/VH/EVs	Slow release	Successful loading of epidermal stem cell exosomes overexpressing VH298 protein in GelMA	Promotes tissue regeneration	[155]
Exos/PF-127	Slow release	HucMSC-exos was added to PF-127 hydrogel	Promoting tissue in regeneration	[138]
Exo-gel	Slow release	Hydrogels with exo release capability formed by the reaction of Tris aminefunctionalized and *N*-hydroxysuccinimide functionalized poly	Anti-inflammatory, promotes tissue regeneration	[166]

*EACPA* epigallocatechin-3-gallate and 3-acrylamido phenylboronic acid complex-based polyacrylamide hydrogel, *EGCG* epigallocatechin-3-gallate, *APBA* 3-acrylamido phenylboronic acid, *Gel-Cip* ciprofloxacin-loaded polydopamine nanoparticles and glycol chitosan hydrogel, *Cip* ciprofloxacin, *PDANPs* polydopamine nanoparticles, *HGGCD* hyaluronic acid methacryloyl and gelatin methacryloyl with graphene oxide quantum dots doping, *AH-3* polyvinylpyrrolidone acrylamide 1-vinyl-3-butylimidazolium and polyethylene glycol dimethacrylate hydrogel, *GP^Gel^* glycopeptide hydrogel, *starPEG-GAG* star-shaped polyethylene glycol and glycosaminoglycan, *MCP-1* monocyte chemoattractant protein–1, *IL-8* interleukin-8, *Gel@fMLP/SiO_2_-FasL* formyl-met-leu-phe and FasL-conjugated silica nanoparticles hydrogel, *fMLP* formyl-met-leu-phe, *SiO_2_-FasL* FasL-conjugated silica nanoparticles, *SF/GA/ZN* Zn^2+^-induced self-assembly of glycyrrhizic acid and silk fibroin hydrogel, *GA* glycyrrhizic acid, *Cu5.4O@Hep-PEG* amine-functionalized star-shaped polyethylene glycol and heparin with Cu5.4O ultrasmall nanozymes, *CIOS/Hydrogel/FGF* hydrogel loaded with chitosan and fibroblast growth factor, *bFGF-HDC@Fe_3_O_4_* fibroblast growth factor-loaded Fe_3_O_4_ nanoparticle, *JNF-A* Janus nanofibrous aerogel, *CS/PEG2* chitosan and prostaglandin 2 hydrogel, *G-DLPUs* L-arginine-based degradable polyurethane and GelMA hydrogel, *ADAS/biomaterials* Cryogel/hydrogel biomaterials loaded with adipose-derived adult stem cells, *CBP/GMS@Cel&INS* a glucose and Matrix Metalloproteinase-9 (MMP-9) dual-response temperature-sensitive shape self-adaptive hydrogel, *HA@MnO_2_/FGF-2/Exos* a hydrogel loaded with manganese dioxide nanosheets, fibroblast growth factor 2 and exosomes, *FHE@exo* Pluronic F127, oxidative hyaluronic acid and EPL hydrogel loaded with adipose mesenchymal stem cells-derived exosomes, *PUAO-CPO-EXO* polyurethane-based oxygen releasing antioxidant scaffolds loaded with exosomes, *Gel/VH/EVs* GelMA hydrogel containing VH298 and human umbilical vein endothelial cells-derived exosomes, *Exos/PF-127*, Pluronic F-127 hydrogel loaded with human umbilical vein endothelial cells-derived exosomes, *Exo-gel* hydrolytically degradable polyethylene glycol hydrogels loaded with macrophages derived exosomes

In recent decades, tremendous progress has been made in the research of biomaterials based on wound healing, which has contributed greatly to the application of biomaterials in the field of tissue engineering [64–67]. Currently, a significant portion of biomaterial research is focusing on various surface-modification systems for modulating the wound microenvironment. Materials for wound repair are generally not limited to one stage of wound healing; however, the material design is based on the different stages of wound healing, and it is also important to consider how different materials modulate properties at different stages of the wound. Wound repair can be promoted by changing the physical properties of the material that carries the physical signals. However, it is important to consider that a material can carry a variety of signals, including chemical and biological signals. Biosignals are favoured by researchers owing to their excellent biosafety. In addition, biomaterials can act as regulatory molecules to modulate the response of the body, although further exploration is needed.

Various antimicrobial dressings have been extensively studied to promote wound healing. However, comparing dressings is difficult because dressings with different structures cannot simultaneously meet the requirements of different wounds, which requires researchers to study the wound-healing effects of dressings more specifically. In addition, the inevitable use of organic solvents and cross-linking agents in the preparation of dressings with specific morphologies necessitates a critical evaluation of material biocompatibility before clinical application. Finally, the development of high-volume production facilities and timely and effective antimicrobial biomedical materials is important for promoting wound healing. This necessitates the use of cost-effective materials and relatively simple synthetic processes to manufacture dressings.

### Biomedical materials for anti-inflammatory treatment

Inflammation is a tightly regulated process that aims to eliminate pathogenic damage by removing damaged tissues and restoring tissue homeostasis [68,69]. Uncontrolled or excessive inflammation can delay wound healing [70]. Macrophages and associated inflammatory factors play important roles in the onset, progression and regression of inflammation [71–73]. After injury, macrophages switch from an M1 (pro-inflammatory) to an M2 (anti-inflammatory) phenotype. Therefore, controlling macrophage polarization has emerged as an attractive approach in regenerative medicine.

Based on their physical structure and ability to bind various bioactive molecules, biomaterials have been developed and used to modulate macrophage function during the inflammatory phase of wound healing, thereby locally manipulating the inflammatory response [12]. In general, most approaches aim to use modified biomaterials to bias macrophage recruitment in wounds toward M2 rather than M1 macrophages [74]. In the inflammatory phase of the wound, biomaterials modulate macrophages in three main ways: (1) slowing the release or conveying appropriate molecules to modulate macrophage polarization; (2) changing the properties of the biomaterials themselves to affect macrophage polarization; or (3) modulating relevant signals in the wound microenvironment.

#### Sustained release strategies

**Figure 3:**
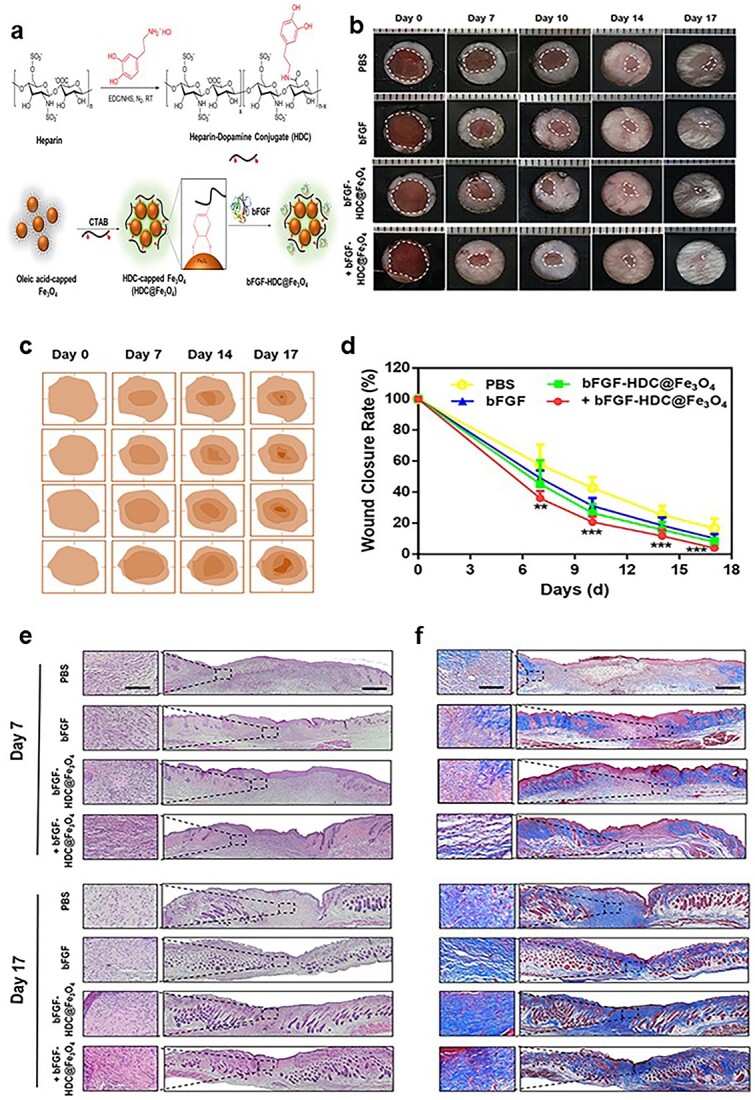
Fe_3_O_4_ nanoparticles loaded with fibroblast growth factor (bFGF) can significantly promote wound healing. (**a**) Schematic illustration of Fe_3_O_4_ nanoparticles loaded with bFGF. (**b**) Representative images of wound beds at different time points after various treatments. (**c**) Wound simulation diagram after different treatments. (**d**) Statistical summary of wound healing rates after different treatments.^*^p < 0.05, ^*^^*^p <0.01, ^*^^*^^*^p < 0.001. (**e**) Representative images of haematoxylin and eosin (H&E) staining of wound tissues that harvested at days 7 and 17 after different treatments. Scale bars: 500 μm for low magnification and 100 μm for high magnification. (**f**) Masson’ s trichrome staining (MTS) of wound tissues that harvested at days 7 and 17 after different treatments. [75] Copyright 2021, ACS Publications

A slow and sustainable local release of biological anti-inflammatory substances can significantly improve wound healing. Compared with conventional scaffold materials, biocompatible nanoparticles can load large amounts of drugs and maintain drug activity. Researchers have developed superparamagnetic iron oxide (Fe_3_O_4_) nanoparticles loaded with fibroblast growth factor (bFGF) using a simple mussel-inspired surface immobilization method that stabilizes bFGF under various conditions and exhibits sustained release of bFGF. *In vivo* and *ex vivo* studies have demonstrated that Fe_3_O_4_ nanoparticles loaded with bFGF can significantly promote wound healing by polarizing M2 macrophages and promoting cell proliferation (Figure 3) [75].

Glycyrrhizic acid (GA) is a natural compound derived from the roots of liquorice plants that has the potential to be an ideal building block for advanced hydrogel dressings with intrinsic immunomodulatory activity. GA and its derivatives have previously been used to treat chronic inflammatory conditions. In one study, hydrogels were loaded with GA that was cross-linked with Zn^+^ and serine proteins to form a dual-network hydrogel that could modulate macrophage polarization and promote wound repair in diabetes [76].

In addition, temperature-sensitive chitosan (CS) hydrogels have been used to load prostaglandin E2 (PGE2) bio-signals with pro-inflammatory immunomodulatory effects [77]. The CS + PGE2 experimental group effectively reduced pro-inflammatory marker expression, increased anti-inflammatory marker production, decreased the proportion of M1-type macrophages among mature macrophages and effectively suppressed the inflammatory response on traumatic surfaces.

#### Modification of internal properties

There are still challenges to overcome in the field of tissue repair and regeneration, including complex material fabrication, uncontrolled drug release, high costs and drug-related side effects; however, various drugs, cytokines and cell delivery in hydrogels are helping to advance the field [78–80]. Recently, researchers have altered the properties of biomaterials in order to directly affect the inflammatory response to trauma, thereby promoting tissue reconstruction [81]. The researchers discovered a glycopeptide compound with a chemical composition similar to that of natural glycoprotein molecules as a base material, and glucomannan was used to form a new type of glycopeptide that assembles into β-type lamellar structures by non-covalent bonding forces and absorbs a large amount of water to form an internal fibrous hydrogel; this material is similar to the skin extracellular matrix, has good biocompatibility and can regulate the inflammatory response, promote vascular renewal and enhance wound repair.

It has also been shown that when the surface groove of the material is 1–5 μm, it is difficult to induce an inflammatory response in the body to regulate the conversion of macrophages to the M2-type, whereas when the surface of the material is sharp, it is easy to induce an inflammatory response in the body [82]. Thus, biomaterials with sharp surfaces may be more conducive to wound healing. In addition, the geometry of 3D scaffolds is related to the modulation of macrophage phenotype. Researchers 3D printed chitosan and poly-L-lactic acid scaffolds with different geometries and pore structures (orthogonal vs. diagonal pores) and found that 3D-printed chitosan-containing scaffolds with diagonal geometries induced the M2 polarization of macrophages [83]. By modifying the inherent properties of the biomaterial, the physical signals carried by the material can better suppress inflammation and promote wound repair.

**Figure 4:**
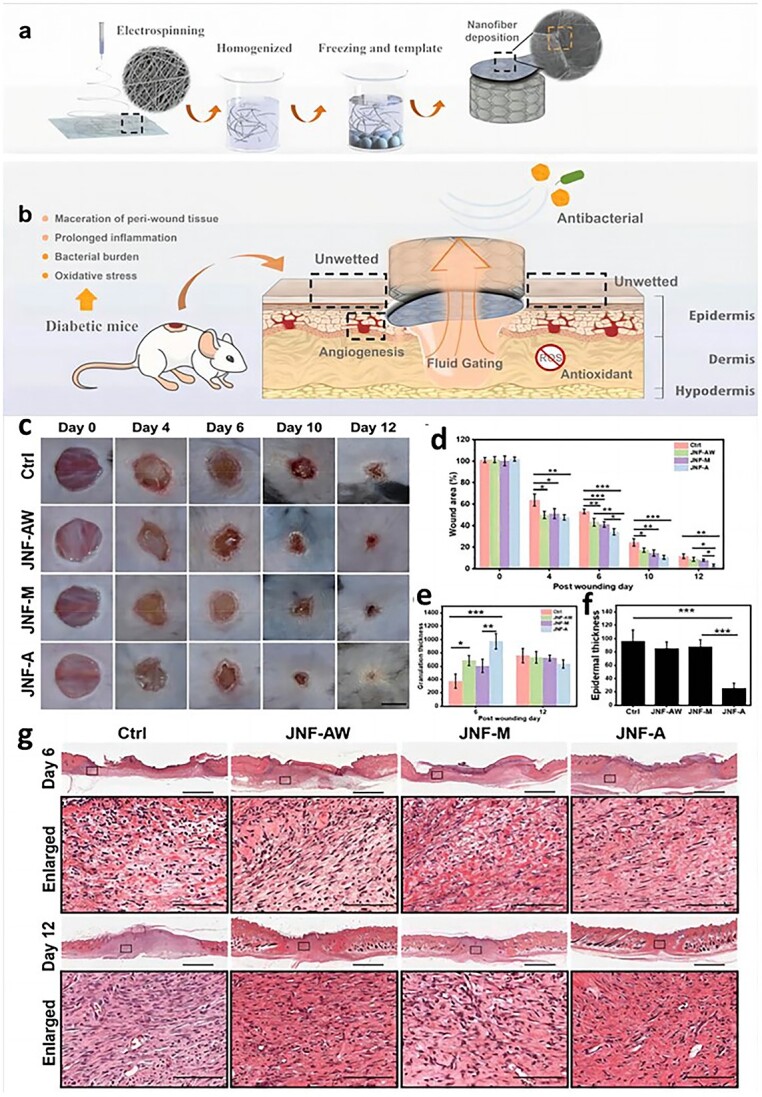
Nanofibrous composite aerogel promotes diabetic wound healing. (**a**) Schematic illustration of the development process of QCS/PVA-PCL/Cur Janus nanofibrous aerogel. (**b**) Diabetic wound treated with the Janus nanofibrous aerogel. (**c**) Representative images of wound healing after being treated with JNF-A, JNF-M, JNF-AW and medical gauze. (**d**) Wound area was measured at different time points. ^*^p < 0.05, ^*^^*^p <0.01, ^*^^*^^*^p < 0.001.(**e**) Quantification of the granulation thickness of regenerated tissue on days 6 and 12. ^*^p < 0.05, ^*^^*^p <0.01, ^*^^*^^*^p< 0.001.(**f**) Quantification of epidermal thickness of regenerated tissue on day 12.^*^p < 0.05, ^*^^*^p<0.01, ^*^^*^^*^p< 0.001. (**g**) Haematoxylin and eosin (H&E) staining for regenerated tissue after being treated with JNF-A, JNF-M, JNF-AW and medical gauze on days 6 and 12. Scale bar: 1 mm (the enlarged images in the box area; scale bar: 200 μm [90] Copyright 2021, Elsevier Ltd. *ROS* reactive oxygen species

Hydrophilic material surfaces are less likely to induce inflammatory responses [84]. Traditional exudate-management dressings rely on hydrophilic materials such as hydrogels, hydrophilic nanofibers and cellulose sponges to absorb and retain exudates [85–88]. However, conventional moist hydrophilic dressings are in close contact with the wound for a long time, and the excess exudate remaining in the dressing can reverse penetration, overhydrate the wound and damage the periwound tissue, thus delaying the healing process [89]. To address these problems, a nanofibre aerogel based on a hydrophobic liquid with unidirectional flow was designed. The authors used a hydrophilic quaternized chitosan and polyvinyl alcohol solution for electrospinning to disperse the nanofibres in anhydrous *tert*-butyl alcohol, lyophilized them to obtain an aerogel, and heat-treated them to stabilize the structure, after which a hydrophobic layer was electrospun on top. The aerogel accelerates diabetic wound healing by synergistically shortening the inflammation period (Figure 4) [90]. Designing materials based on physical signals that can be modified according to the pathological features of wound healing requires a deeper understanding of the specific wound-healing mechanisms.

#### Effective microenvironmental signal stimulation

During the inflammatory phase of the wound, local tissue blood vessels are constricted and the blood supply is insufficient, resulting in a lack of nutrition and oxygen and an increase in glycolysis, which raises lactic acid and PaCO_2_ levels while reducing the local pH of the wound. At the site of wound inflammation, neutrophils, endothelial cells and senescent fibroblasts can produce large amounts of reactive oxygen species (ROS) and release them into the inflamed tissue. Microenvironmental ROS selectively leads to the activation of transcription factors that control the expression of pro-inflammatory cytokines (interleukin-1, interleukin-17 and Tumor necrosis factor-α (TNF-α)), chemokines and protein hydrolases, including Matrix Metalloproteinases (MMPs) and serine proteases.

**Figure 5:**
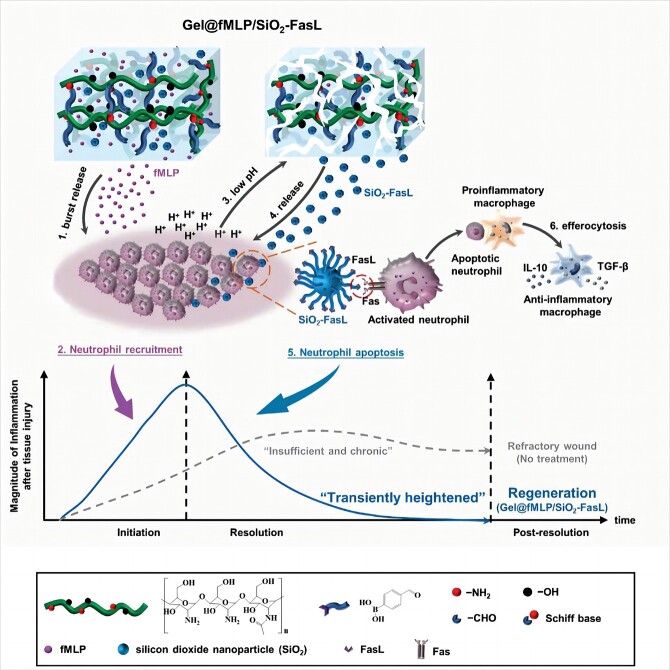
Schematic illustration of the Gel@fMLP/SiO2-FasL for transiently heightened inflammatory response manipulation to initiate refractory wound healing. [93] Copyright 2022, Advanced Science (Weinh)

Biomaterials can help suppress inflammation and promote wound repair based on the wound microenvironment. Activation of the Fas signalling pathway has been shown to induce neutrophil apoptosis, and accelerated phagocytosis of apoptotic neutrophils by macrophages can activate key intracellular signals, thus promoting macrophage phenotype transformation [91,92]. Therefore, a pH-responsive hydrogel composed of formyl-met-leu-phe and FasL-coupled silica nanoparticles (SiO_2_-FasL) was designed (Figure 5). Formyl-met-leu-phe can recruit neutrophils and enhance the early effects of inflammation. Following acid production by neutrophils, the hydrogel degrades, exposing it to SiO_2_ and FasL. The FasL carboxyl group can bind to the SiO_2_ amino group, and FasL can bind to Fas on the surface of target cells to initiate apoptosis and ultimately promote macrophage polarization and phenotype transformation [93]. To promote diabetic wound repair, ROS-responsive polyvinyl alcohol hydrogels have also been developed for the unique wound microenvironment [93, 94].

Although none of the methods discussed above have been explicitly tested in clinical settings for trauma healing, they offer various novel approaches for non-invasive local control of the inflammatory response. However, there is still much to learn about optimally manipulating the inflammatory response.

### Biomedical materials for antibacterial treatment

Neutrophils are recruited to the wound in the early stages of acute and chronic wound healing to destroy planktonic bacteria and prevent the formation of bacterial biofilms [95]. This behaviour is dependent on fibroblasts secreting various cytokines; however, chronic inflammation in the wound microenvironment caused by oxidative stress, impaired angiogenesis and bacterial infection can impair the ability of neutrophils to prevent biofilm formation [96–98]. Bacteria produce biofilms from polysaccharides to protect themselves from drug treatment and neutrophils, thereby extending the wound-healing process [99,100]. In patients with burns, infection is responsible for 75% of all deaths [101]. Consequently, an increasing number of researchers have designed numerous biomedical materials to reduce the risk of bacterial infection in wounds [102–104].

#### Antimicrobial signal delivery strategies

The main methods of designing common antimicrobial materials are to use the material as a carrier to carry or immobilize an antimicrobial signal, or to use the material’s intrinsic properties to remove bacteria. Common antimicrobial signals include antibiotics, antimicrobial peptides, chitosan, curcumin, quaternary ammonium salts, nanoparticles and cationic organic agents [105–107].

Antibiotics have been used to treat pathogenic bacterial strains since the 1940s [96,98–101,103–105,108]. Antibiotics have been widely used against bacteria and have successfully treated many infections; however, antibiotic misuse persists, and there are no uniform criteria for evaluating controlled release of drugs to ensure optimal therapeutic efficiency. Several researchers have recently developed synthetic materials with intelligent local slow-release antibiotic capabilities [109]. Gao *et al*. reported a ciprofloxacin-loaded polydopamine glycol chitosan hydrogel; the hydrogel composite system significantly inhibited the biological activity of *Staphylococcus aureus* and *Escherichia coli* after near-infrared (NIR) light illumination owing to the photothermal effect of polydopamine nanoparticles, and was able to not only synergistically sterilize but also achieve precise release of antibiotics after NIR light illumination [110].

The broad-spectrum antimicrobial activities of cationic polymers and nanoparticles make them popular among researchers. Pathogens can be effectively inactivated by electrostatic interactions between positively charged polymers and negatively charged bacterial cell membranes. Cationic polymers contain networks of positively charged groups, can be composed of natural or synthetic organic polymers and have been used to construct antimicrobial biomaterials [111]. For example, using polyvinylpyrrolidone acrylamide, 1-vinyl-3-butylimidazole and polyethylene glycol dimethacrylate, Wang *et al*. prepared a novel antimicrobial hydrogel wound dressing [112]. The hydrogel contained cationic groups in the polymer chain, which resulted in excellent antibacterial activity; *in vivo* experimental results demonstrated that the hydrogel effectively promoted the wound-healing process.

Several nanoparticles have been studied for potential antimicrobial applications, including those containing silver, copper, gold and other metals [113,114]. However, the use of inorganic nanoparticles as antimicrobial agents is hampered by their nonspecific biotoxicity and immune system effects, limiting their clinical utility. Therefore, the application of these nanoparticles should be achieved by incorporating specific formulations.

Recently, antimicrobial peptides (AMPs), which are non-toxic and non-resistant small-molecule peptides, have been proposed for treating drug-resistant bacterial strains [115–119]. Researchers grafted AMPs onto elastin-like polypeptide surfaces, resulting in a significant bactericidal effect on *S. aureus* [120]. However, the application of natural AMPs is limited because of their high production costs and environmental sensitivity. Inspired by natural AMPs, amphiphilic polymers composed of cationic and hydrophobic residues have been designed to assemble polymers as bioactive reagent delivery systems with inherent antimicrobial properties, and well-designed synthetic AMPs can achieve better targeting efficiency. However, more effort is required to achieve controlled synthesis of AMPs with mass production for a wide range of applications.

#### Surface modifications for antimicrobial treatment

In addition to a material carrying antimicrobial agents to achieve bactericidal effects, the material itself can be modified to induce bacterial cell membrane rupture by allowing the pathogen to come into direct contact. For example, graphene oxide nanosheets can use the ‘nanoblade effect’ to break the lipid bilayer of bacteria [121].

In addition to their unique morphology, the wings of cicadas and dragonflies exhibit antimicrobial activity because of the nanoscale columnar structures on their surfaces. Inspired by their unique structures, several synthetic bio-nanomorphic surfaces with similar nanomorphologies have been used for effective bacterial killing [122]. Other researchers have designed a surface microstructure based on shark skin that disrupts the formation of *S. aureus* biofilms without using biocides, resulting in a 50–80% reduction in colonization rates [123]. The antimicrobial effect of these biological surfaces is primarily due to their effect on bacterial adhesion; the microstructures and nanostructures on the material surface reduces the contact area with bacteria, resulting in a reduction of force between the bacteria and the material.

Femtosecond lasers have also been used to produce nanostrip arrays and nanoweb structures on the surface of stainless steel [124]. Both nanostructures were found to reduce *S. aureus* adhesion on the stainless steel surface, but the nanoweb structure was superior to the nanostrip array structure in terms of antibacterial adhesion performance. The hydrophobic properties of this material can also be used to create antibacterial agents [125].

Furthermore, superhydrophobic surfaces can inhibit bacterial adhesion owing to the mutual repulsion between the surface and the hydrophobic surface of bacteria. Bacterial contact and adhesion are reduced on superhydrophobic surfaces because of the adsorption of an air film on the surface, which reduces the solid area fraction, resulting in a self-cleaning ability of the surface, which can effectively remove initially-adhered bacteria in subsequent cleaning. However, these changes in the physical signals of the material have limited bactericidal effects.

The recent development of photothermal therapy (PPT) and photodynamic therapy (PDT) has resulted in significant advances in biomaterials for antimicrobial purposes. PPT is a safe and effective strategy for combating infections using appropriate photothermal materials that can convert light into heat and kill bacteria via cell wall degradation and protein/enzyme denaturation. For example, in one study, a polymer substrate immersed in a weakly alkaline dopamine solution resulted in a polydopamine photothermal antimicrobial coating on its surface that exhibited excellent photothermal antibacterial properties against *S. aureus*, *E. coli* and *Candida albicans* under NIR light (808 nm, 0.9 W·cm^−2^) [126]. PPT is based on the heat generated by nanopreparation when exposed to NIR light (700–1400 nm) irradiation. NIR light penetrates deeper into tissue while causing no significant damage to normal tissue, allowing for precise remote control of the bactericidal treatment. Fullerenes, which have semiconductive and ROS-generating properties, could improve the antibacterial activity of these drugs [121,127].

PDT is another promising strategy for precisely treating persistent bacteria remotely. PDT requires three factors: photosensitizers (PS), light sources and oxygen. When PS are irradiated with light at a wavelength matching the maximum absorption, the excited-state PS can generate toxic ROS, including superoxide, singlet oxygen (^1^O_2_) and hydroxyl radicals. The generated ROS can damage pathogen cell membranes and DNA [119,128,129].

However, eliminating the potential toxicity of photothermal and photosensitive materials remains challenging. Furthermore, the inconvenience of light intervention during treatment, damage to normal tissues and low efficiency make the application of precise local light-assisted therapy difficult.

### Biomedical materials for regeneration

Proliferation and remodelling are important stages in the healing of skin wounds, and chronic wounds may require a longer healing period owing to persistent inflammation, bacterial infection and abnormal growth factor reduction. The presence of hyperglycaemia in diabetic wounds makes the formation of new blood vessels difficult, thus limiting oxygen and nutrient access to the wound site [130]. Consequently, several substances may need to be delivered exogenously to promote wound healing. These substances include growth factors, natural proteins, engineered peptides, stem cells and exosomes [131–143]. In most cases, these substances require repeated use at high doses to be effective and proteases in cells degrade them. Therefore, researchers have used biomaterials to construct various delivery systems that maintain therapeutic substance activity by precise control and sustained release at trauma sites by constructing biomaterials with scaffolding characteristics to slowly release active substances.

Some biomaterials aid in wound regeneration; in many cases, the material is used as a slow-release vehicle to combine the relevant active substance with the material, resulting in its controlled release. There are also ways to achieve slow release in the wound using degradable biocompatible materials combined with an active substance. L-Arginine can be converted into nitric oxide and ornithine via nitric oxide synthase. Nitric oxide promotes angiogenesis, whereas ornithine induces proline synthesis. Proline is a substrate for collagen, which promotes wound healing. However, excess L-arginine in wounds can affect the activity of ornithine decarboxylase, resulting in scarring and fibrosis [143–147]. Therefore, researchers have used carboxyl-rich levulinic acid-modified polyurethane and gelatin methacryloyl to form a dual network to achieve a slow release of levulinic acid from wounds and successfully promote wound healing [148].

The characteristics of the wound microenvironment can also be used to achieve smart release of an active substance. For example, researchers have designed a dual-responsive temperature-sensitive hydrogel that successfully promotes the healing of diabetic wounds. Phenylboronic acid was grafted on top of chitosan, which forms a reversible chemical bond by combining the ester bond of phenylboronic acid chitosan with the hydroxyl group of polyvinyl alcohol and wrapping insulin in it. It is easier to combine the phenylboronic acid ester group with the hydroxyl group of glucose than with the hydroxyl group of polyvinyl alcohol because of the high-glucose environment in diabetic wounds. This breaks the ring of the composite hydrogel, leading to a highly glucose-responsive insulin release [149]. The design of sustained-release materials provides new ideas for future sustained-release designs.

To achieve a satisfactory repair effect, an exogenous active substance must be selected and an intelligent slow-release method must be designed. Recently, exosomes have emerged as an exciting research topic in the field of regeneration [150–152]. However, using exosomes for wound treatment is challenging because they can be rapidly removed from the wound site and only survive in the body for a short period [153]. Therefore, the combination of exosomes with biomaterials that can prolong their retention time at the wound site, as well as wound dressings that do not affect their biological activity, has become the focus of research for the development of exosome-based therapies that synergistically promote chronic wound healing and complete skin regeneration [141, 154]. Researchers have also designed a PluronicF-127 hydrogel with slow-release human umbilical cord-derived mesenchymal stem cell-derived exosomes, which greatly facilitates skin wound healing [142].

**Figure 6:**
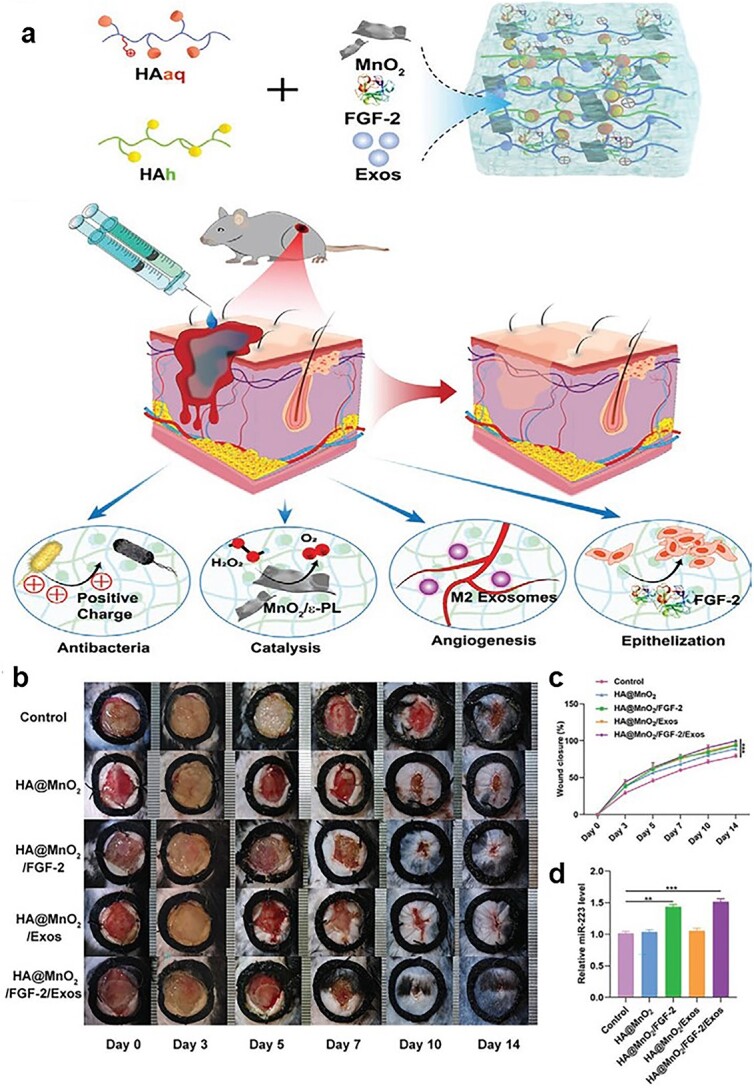
Multifunctional hydrogel including exosome and fibroblast accelerates oxidative diabetic wound healing. (**a**) Construction and application of the multifunctional HA@MnO2/FGF-2/Exos hydrogel. (**b**) Representative images of the wound healing process of mice treated with PBS, HA@MnO2 hydrogel, HA@MnO2/FGF-2 hydrogel, HA@MnO2/Exos hydrogel and HA@MnO2/FGF-2/Exos hydrogel. (**c**) *In vivo* wound closure rates of the five groups at different time points.^*^p < 0.05, ^*^^*^p <0.01, ^*^^*^^*^p < 0.001. (**d**) On day 14 post-wounding, skin tissues were collected from each of the five groups and the level of miR-223 was assessed using a Real time quantitative polymerase chain reaction(RT-qPCR) analysis. ^*^p < 0.05, ^*^^*^p <0.01, ^*^^*^^*^p < 0.001. [154] Copyright 2021, Small. *HA@MnO2/FGF-2/Exos* a hydrogel loaded with manganese dioxide nanosheets, fibroblast growth factor 2 and exosomes

However, wound pathogenesis is complex, and biomaterials carrying only simple exosomes have limited therapeutic effect. A synergistic approach can significantly accelerate wound healing. Researchers created a composite hydrogel composed of antioxidant polyurethane and exosomes from adipose-derived stem cells that can simultaneously reduce oxidative stress, provide oxygen and induce angiogenesis to promote diabetic wound healing [155]. Xiong *et al*. encapsulated both M2-derived exosomes and FGF-2 in a hydrogel that synergistically promoted diabetic wound healing (Figure 6) [156].

In addition to encapsulating multiple therapeutic agents in hydrogels to achieve therapeutic effects, the pathological mechanisms of wound healing can be targeted by designing responsively-engineered exosomes and utilizing the slow-release properties of biomaterials to promote wound healing. Endothelial dysfunction is a cause of impaired angiogenesis in patients with diabetes [157]. VH298 is a small molecule compound that stabilizes hypoxia-inducible factor-1α (HIF-1α). Under normoxic conditions, hydroxylated HIF1-α is specifically recognized by von Hippel–Lindau tumour suppressor protein, which recruits ubiquitinating enzymes, leading to the degradation of HIF-1α by the ubiquitin proteasome [158]. One study prepared an epidermal stem cell-derived exosome hydrogel loaded with overexpressed VH298 protein, which significantly promoted wound healing by activating the HIF-1α signalling pathway [159]. The slow release of exosomes still needs to be explored, and hydrogels with genetically engineered exosomes are expected to become a new research trend in the future.

### Future perspective

#### Advances in research on biomaterials for wound healing

Wound healing is a complex process, especially in chronic wounds such as diabetic foot trauma ulcers, where the slow healing rate is one of the most serious problems that threaten the health of millions of people. To overcome this challenge, extensive research on biomaterials, combining different types of bioactive molecules, has led to the development of intelligent biomaterials. Smart biomaterials are inherently attractive due to their various advantages. Recent findings have increasingly shown the great potential of guided inflammation, infection and regeneration in improving wound healing. In these respects, an increasing number of biomaterial design strategies are being proposed. Biomaterials are often designed so that multiple substances can be released in combination at different stages of wound healing, thereby accelerating the process. Overall, the future may lie in the development of new biomaterials with multiple roles (including improving hypoxia, enhancing angiogenesis, reducing oxidative stress and preventing infection) that may modulate wound healing at all stages and provide a balanced environment throughout the wound healing process, thereby reducing potential complications. Many of the therapeutic effects of biomaterials utilize a complex of materials with different functions that act on different stages of wound healing, thus achieving a synergistic promotion of the process.

#### Trends in material design for the trauma healing phase

In addition to macrophages, T cells play an important role in regulating the inflammatory response during the inflammatory phase of trauma repair. γδ T cells in the skin have been shown to provide the necessary cytokines and growth factors that regulate intraepidermal homeostasis, but also promote inflammation at the wound site. Dermal γδ T cells provide cytokines and chemokines that regulate inflammation throughout the skin and feed back to epidermal γδ T cells [160,161]. Given this, epidermal and dermal γδ T cells promote complex crosstalk with keratin-forming cells and inflammatory cells to provide homeostasis and maintain skin homeostasis. Therefore, future material design strategies could consider targeting more immune cells in the skin to be able to better control inflammation. Slowing the release of responsive antimicrobial substances and improving surface signalling of biomaterials can effectively reduce microbial colonization of traumatic surfaces. The design of cost-effective materials and antimicrobial biomaterials with simple synthesis processes remains a problem for researchers to address. In the proliferation and remodelling phase of trauma healing, material-loaded exosomes for trauma treatment will become a new therapeutic trend as the effectiveness of exosomes in trauma repair has been well proven; therefore, improving the slow-release efficiency of exosomes and finding more cost-effective ways of engineering exosomes are still issues that need to be addressed in the future.

#### Problems faced by biomaterials governance in clinical and practical applications

There are many requirements for the design of materials to promote wound healing, such as the need for good adhesion and haemostatic properties. Recently people are also investigating the wound detection functions of hydrogels, such as can be achieved by adding some conductive biomaterials to the hydrogel [162–164]. Such biomaterials can respond to the healing of deep wounds by monitoring the strength of the current, thus providing on-demand treatment. However, antibacterial, anti-inflammatory and regeneration are the main ways to promote wound healing, but these phases are not separate, which requires researchers to make good use of biomedical materials to achieve synergistic promotion of wound healing.

## Conclusions

This paper reviews the design approaches of biomedical materials for anti-inflammatory, antibacterial and regeneration promotion. Anti-inflammation is an important strategy in the inflammatory phase of wound healing, and the design of anti-inflammatory biomaterials must include the slow release of relevant molecules, changing the physicochemical properties of the biomaterials or using biomaterials to mediate relevant signals in the trauma microenvironment. This in turn leads to the conversion of macrophages in the trauma into M2 type macrophages, and future research should also consider using other immune cells to achieve better control of inflammation. Antimicrobials are also an important tool in improving healing efficiency. The key to the design of common antimicrobial materials is to use the material as a carrier to carry/immobilize the antimicrobial signal; bactericidal effects can also be achieved using the material’s own properties through physical/chemical modifications of the surface of the material. The synthesis of more efficient and economical antimicrobial materials is a problem that needs to be addressed in the future. The key to accelerating the proliferation and remodelling phase of wound healing is to use the carrier slow-release properties of biomaterials to provide the relevant proliferation and regenerative active molecules to synergistically promote wound repair. The choice of relevant active molecules largely affects the efficiency of wound healing and so exosomal synergistic biomaterial therapy has become a new therapeutic trend.

## Data Availability

The data used in this publication are available upon request.

## Abbreviations

AMPs: Antimicrobial peptides; bFGF: Fibroblast growth factor; Fe_3_O_4_: Superparamagnetic iron oxide; GA: Glycyrrhizic acid; HIF-1α: Hypoxia-inducible factor-1α; NIR: Near-infrared; PDT: Photodynamic therapy; PGE2: Prostaglandin E2; PPT: Photothermal therapy; PS: Photosensitizers; ROS: Reactive oxygen species.
